# Supplementary material for: Socioeconomic Deprivation and Invasive Breast Cancer Incidence by Stage at Diagnosis: A Possible Explanation to the Breast Cancer Social Paradox
Source: Cancers (Basel). 2024 Apr 27;16(9):1701. doi: 10.3390/cancers16091701 (PMC11083525; doi:10.3390/cancers16091701)

SUPPLEMENTARY MATERIALS

Supplementary Table S1. Characteristics of breast cancer cases excluded from the study according to exclusion criteria.

**Characteristics**	**Missing TNM stage**	**Missing F-EDI**	**Missing F-EDI and TNM stage**
	N (%)	N (%)	N (%)
**Cancer cases**	1,088	323	5
**Age** (y/o)			
15-49	185 (17%)	58 (18%)	0 (0%)
50-74	283 (26%)	188 (58%)	1 (20%)
75+	620 (57%)	77 (24%)	4 (80%)
**Rurality of residence**			
Rural	265 (24%)	0 (0%)	0 (0%)
Urban	823 (76%)	323 (100%)	5 (100%)
**Mode of detection**			
Symptoms	640 (59%)	160 (50%)	3 (60%)
Organized screening	65 (6%)	66 (20%)	0 (0%)
Opportunistic screening	66 (6%)	71 (22%)	0 (0%)
Other	33 (3%)	17 (5%)	0 (0%)
Unknown	284 (26%)	9 (3%)	2 (40%)
**Stage**			
I	-	125 (39%)	-
II	-	118 (37%)	-
III-IV	-	80 (25%)	-
**Socioeconomic deprivation** (F-EDI quintile)			
Q1 The most affluent	215 (20%)	-	-
Q2	226 (21%)	-	-
Q3	224 (21%)	-	-
Q4	210 (19%)	-	-
Q5 The most deprived	213 (20%)	-	-

Supplementary Table S2. Age-standardised incidence rates of breast cancer per 100,000 women-year and 95% confidence intervals according to socioeconomic deprivation (F-EDI quintile) and rurality of the residence, by age group and stage at diagnosis (n=33,298; 2008-2015).

**Age group**		**Stage I**	**Stage II**	**Stage III-IV**	**All-stages**
y/o		ASIR (95% CI)*	ASIR (95% CI)*	ASIR (95% CI)*	ASIR (95% CI)*
**15-49**	Q1	32.9 (30.5-35.4)	27.2 (25.0-29.5)	10.1 (8.7-11.4)	70.2 (66.6-73.9)
	Q2	31.0 (28.6-33.5)	24.0 (21.9-26.1)	11.7 (10.2-13.1)	66.7 (63.1-70.2)
	Q3	30.4 (27.9-33.0)	24.3 (22.0-26.6)	10.2 (8.8-11.7)	65.0 (61.3-68.7)
	Q4	28.0 (25.8-30.2)	25.1 (23.0-27.1)	10.6 (9.2-11.9)	63.7 (60.4-67.0)
	Q5	26.0 (24.0-28.0)	21.6 (19.7-23.4)	9.6 (8.4-10.9)	57.2 (54.2-60.1)
	Urban	30.7 (29.5-31.9)	24.4 (23.3-25.4)	10.3 (9.6-10.9)	65.3 (63.6-67.1)
	Rural	27.6 (25.3-29.9)	25.5 (23.3-27.7)	11.0 (9.6-12.5)	64.1 (60.6-67.6)
	All areas	30.0 (28.9-31.0)	24.6 (23.7-25.6)	10.5 (23.7-25.6)	65.1 (63.5-66.6)
**50-74**	Q1	203.6 (199.5-207.8)	92.9 (90.1-95.7)	39.6 (37.8-41.4)	336.1 (330.8-341.4)
	Q2	188.1 (183.7-192.4)	90.1 (87.1-93.1)	40.5 (38.5-42.5)	318.7 (313.0-324.4)
	Q3	183.5 (178.9-188.2)	93.6 (90.3-96.9)	45.1 (42.8-47.4)	322.2 (316.1-328.3)
	Q4	177.2 (172.7-181.8)	82.5 (79.4-85.6)	44.6 (42.3-46.9)	304.3 (298.3-310.2)
	Q5	163.9 (159.3-168.4)	81.7 (78.5-84.9)	45.5 (43.1-47.9)	291.0 (285.0-297.1)
	Urban	187.1 (184.8-189.4)	87.7 (86.1-89.2)	41.8 (40.7-42.8)	316.5 (313.6-319.5)
	Rural	176.7 (172.6-180.8)	91.6 (88.7-94.6)	46.4 (44.3-48.5)	314.7 (309.2-320.2)
	All areas	184.8 (182.8-186.8)	88.5 (87.2-89.9)	42.8 (87.2-89.9)	316.1 (313.5-318.7)
**75+**	Q1	107.4 (106.4-108.3)	109.3 (108.3-110.3)	74.9 (74.1-75.8)	291.6 (290.0-293.2)
	Q2	101.2 (100.3-102.1)	100.1 (99.2-101.0)	84.6 (83.8-85.5)	285.9 (284.4-287.5)
	Q3	90.9 (90.0-91.8)	94.9 (94.0-95.8)	83.6 (82.8-84.5)	269.4 (267.9-271.0)
	Q4	100.2 (99.3-101.1)	93.4 (92.6-94.3)	86.3 (85.5-87.2)	279.9 (278.4-281.4)
	Q5	96.8 (95.9-97.8)	87.1 (86.2-88.1)	94.2 (93.2-95.1)	278.2 (276.5-279.8)
	Urban	103.1 (102.6-103.5)	97.3 (96.9-97.8)	84.1 (83.6-84.5)	284.5 (283.7-285.3)
	Rural	83.2 (82.3-84.0)	94.1 (93.2-95.0)	88.4 (87.6-89.3)	265.7 (264.2-267.3)
	All areas	99.1 (98.7-99.5)	96.7 (96.3-97.1)	84.9 (96.3-97.1)	280.8 (280.1-281.5)
**15+**	Q1	79.5 (76.6-82.5)	46.7 (44.4-49.1)	19.6 (18.2-21.1)	145.9 (141.9-150.0)
	Q2	73.9 (70.9-76.9)	43.5 (41.1-45.8)	21.3 (19.7-22.9)	138.7 (134.5-142.8)
	Q3	72.1 (68.9-75.2)	44.4 (41.9-46.9)	21.4 (19.8-23.1)	137.9 (133.5-142.3)
	Q4	69.0 (66.0-71.9)	42.0 (39.7-44.4)	21.6 (20.0-23.3)	132.6 (128.5-136.7)
	Q5	63.9 (61.1-66.7)	39.1 (36.9-41.4)	21.4 (19.8-23.0)	124.5 (120.6-128.4)
	Urban	73.5 (72.0-75.0)	43.0 (41.8-44.2)	20.6 (19.8-21.4)	137.1 (135.1-139.2)
	Rural	68.1 (65.2-70.9)	44.7 (42.3-47.1)	22.5 (20.8-24.1)	135.3 (131.2-139.3)
	All areas	72.3 (71.0-73.6)	43.4 (42.3-44.4)	21.0 (42.3-44.4)	136.7 (134.9-138.6)

*F-EDI quintiles: from Q1 the most affluent to Q5 the most deprived

Supplementary Table S3. Incidence rate ratios (IRR) of breast cancer according to socioeconomic deprivation (F-EDI quintile), and rurality of the residence, by age group and stage at diagnosis (n=33,298; 2008-2015).

**Age group**		**Stage I**		**Stage II**		**Stage III-IV**		**All-stages**	
y/o		IRR (95% CI)	**p**	IRR (95% CI)	**p**	IRR (95% CI)	**p**	IRR (95% CI)	**p**
	**F-EDI**		**<0.001**		**0.012**		0.439		**<0.001**
	Q1	1 (reference)		1 (reference)		1 (reference)		1 (reference)	
	Q2	0.94 (0.85 – 1.04)		0.89 (0.79 – 0.99)		1.15 (0.97 – 1.36)		0.95 (0.89 – 1.02)	
	Q3	0.91 (0.82 – 1.01)		0.89 (0.80 – 1.01)		1.02 (0.85 – 1.23)		0.92 (0.85 – 0.99)	
**15-49**[1]	Q4	0.82 (0.73 – 0.91)		0.92 (0.82 – 1.03)		1.06 (0.88 – 1.28)		0.89 (0.83 – 0.96)	
	Q5	0.73 (0.65 – 0.82)		0.80 (0.70 – 0.91)		0.99 (0.81 – 1.21)		0.79 (0.73 – 0.86)	
	**Rurality**		**<0.001**		0.834		0.646		**0.023**
	Urban	1 (reference)		1 (reference)		1 (reference)		1 (reference)	
	Rural	0.85 (0.78 – 0.93)		1.01 (0.92 – 1.11)		1.03 (0.90 – 1.19)		0.94 (0.88 – 0.99)	
	**F-EDI**		**<0.001**		**0.013**		**0.020**		**<0.001**
	Q1	1 (reference)		1 (reference)		1 (reference)		1 (reference)	
	Q2	0.93 (0.88 – 0.99)		0.96 (0.89 – 1.05)		1.02 (0.90 – 1.16)		0.95 (0.90 – 1.00)	
	Q3	0.91 (0.85 – 0.97)		1.00 (0.92 – 1.09)		1.14 (1.01 – 1.29)		0.97 (0.92 – 1.02)	
**50-74**[1]	Q4	0.86 (0.81 – 0.92)		0.89 (0.81 – 0.97)		1.15 (1.01 – 1.31)		0.91 (0.86 – 0.95)	
	Q5	0.78 (0.72 – 0.83)		0.89 (0.81 – 0.98)		1.21 (1.06 – 1.38)		0.86 (0.81 – 0.90)	
	**Rurality**		**0.001**		0.688		**0.009**		0.129
	Urban	1 (reference)		1 (reference)		1 (reference)		1 (reference)	
	Rural	0.91 (0.87 – 0.96)		1.01 (0.95 – 1.09)		1.14 (1.03 – 1.26)		0.97 (0.93 – 1.01)	
	**F-EDI**		0.065		**0.011**		0.102		0.186
	Q1	1 (reference)		1 (reference)		1 (reference)		1 (reference)	
	Q2	0.95 (0.83 – 1.09)		0.92 (0.80 – 1.05)		1.12 (0.96 – 1.32)		0.99 (0.91 – 1.07)	
	Q3	0.86 (0.75 – 0.98)		0.87 (0.76 – 1.00)		1.10 (0.94 – 1.30)		0.92 (0.85 – 1.01)	
**75+**	Q4	0.91 (0.79 – 1.03)		0.84 (0.74 – 0.97)		1.15 (0.98 – 1.35)		0.94 (0.87 – 1.03)	
	Q5	0.83 (0.72 – 0.96)		0.78 (0.68 – 0.90)		1.26 (1.07 – 1.49)		0.91 (0.83 – 1.00)	
	**Rurality**		**<0.001**		0.174		0.112		0.057
	Urban	1 (reference)		1 (reference)		1 (reference)		1 (reference)	
	Rural	0.81 (0.72 – 0.92)		0.92 (0.82 – 1.04)		1.10 (0.98 – 1.25)		0.93 (0.87 – 1.00)	
	**F-EDI**		**<0.001**		**<0.001**		**0.007**		**<0.001**
	Q1	1 (reference)		1 (reference)		1 (reference)		1 (reference)	
	Q2	0.93 (0.89 – 0.98)		0.93 (0.87 – 0.99)		1.08 (0.99 – 1.18)		0.95 (0.92 – 0.99)	
	Q3	0.90 (0.85 – 0.95)		0.94 (0.88 – 1.00)		1.10 (1.01 – 1.20)		0.94 (0.90 – 0.98)	
**15+**[1]	Q4	0.86 (0.81 – 0.91)		0.89 (0.83 – 0.95)		1.13 (1.03 – 1.23)		0.91 (0.87 – 0.95)	
	Q5	0.77 (0.72 – 0.82)		0.84 (0.78 – 0.90)		1.18 (1.08 – 1.29)		0.85 (0.81 – 0.89)	
	**Rurality**		**<0.001**		0.884		**0.002**		**0.008**
	Urban	1 (reference)		1 (reference)		1 (reference)		1 (reference)	
	Rural	0.89 (0.85 – 0.93)		1.00 (0.95 – 1.05)		1.11 (1.04 – 1.19)		0.96 (0.92 – 0.99)	

[1] adjusted by age classes (15-29, 30-39, 40-49, 50-59, 60-64, 65-74 and 75+ y/o)

IRR obtained from Poisson model with nested random at the municipality/IRIS level

F-EDI: from Q1 the most affluent to Q5 the most deprived

Supplementary Figure S1. Age-standardised incidence rates (ASIR) of breast cancer and 95% confidence intervals according to age group and rurality of residence, by stage at diagnosis (n=33,298; 2008-2015).

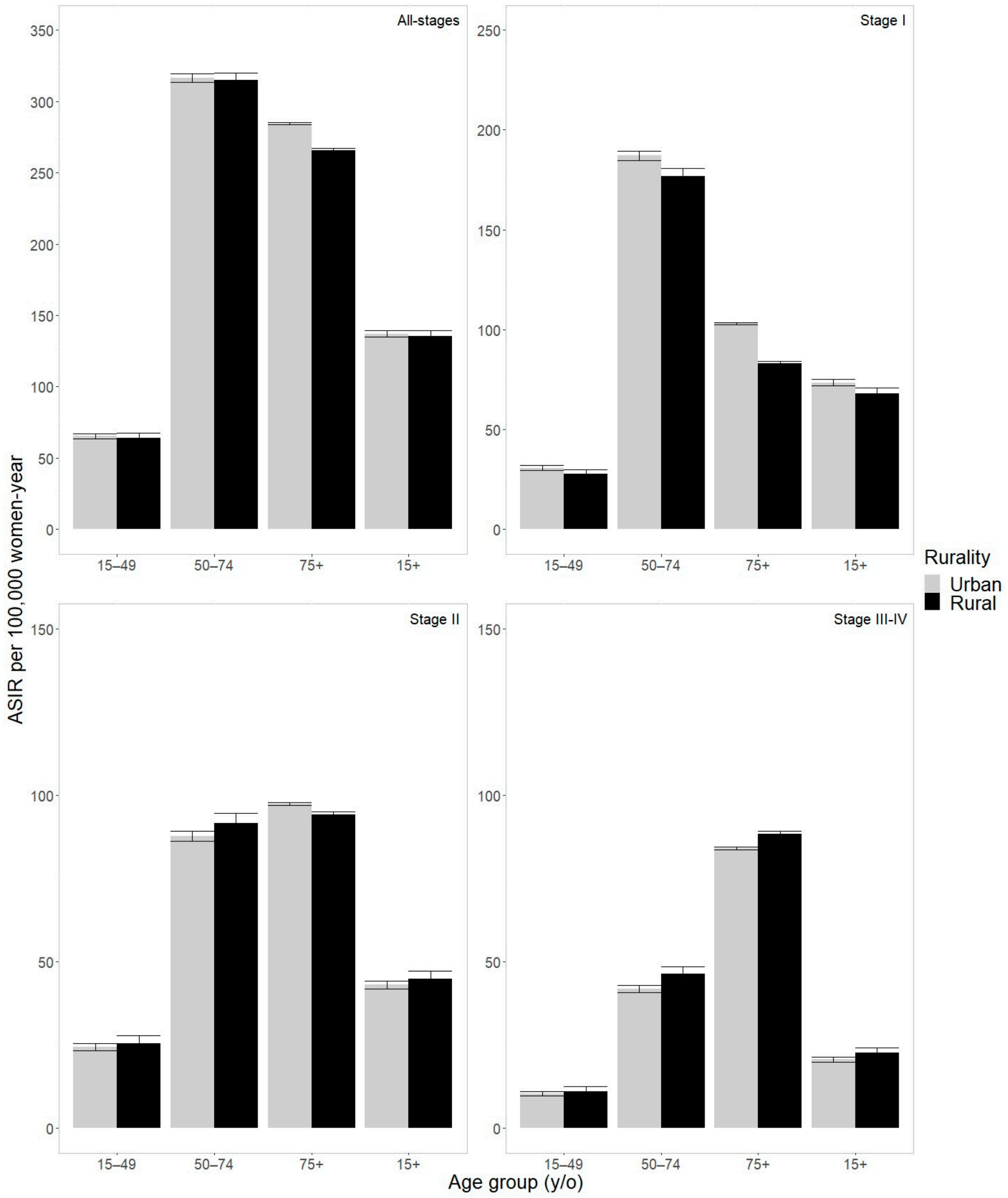

Supplementary Figure S2. Incidence rate ratios (IRR) and 95% confidence intervals according to age group and rurality of the residence, by stage at diagnosis (n=33,298; 2008-2015).

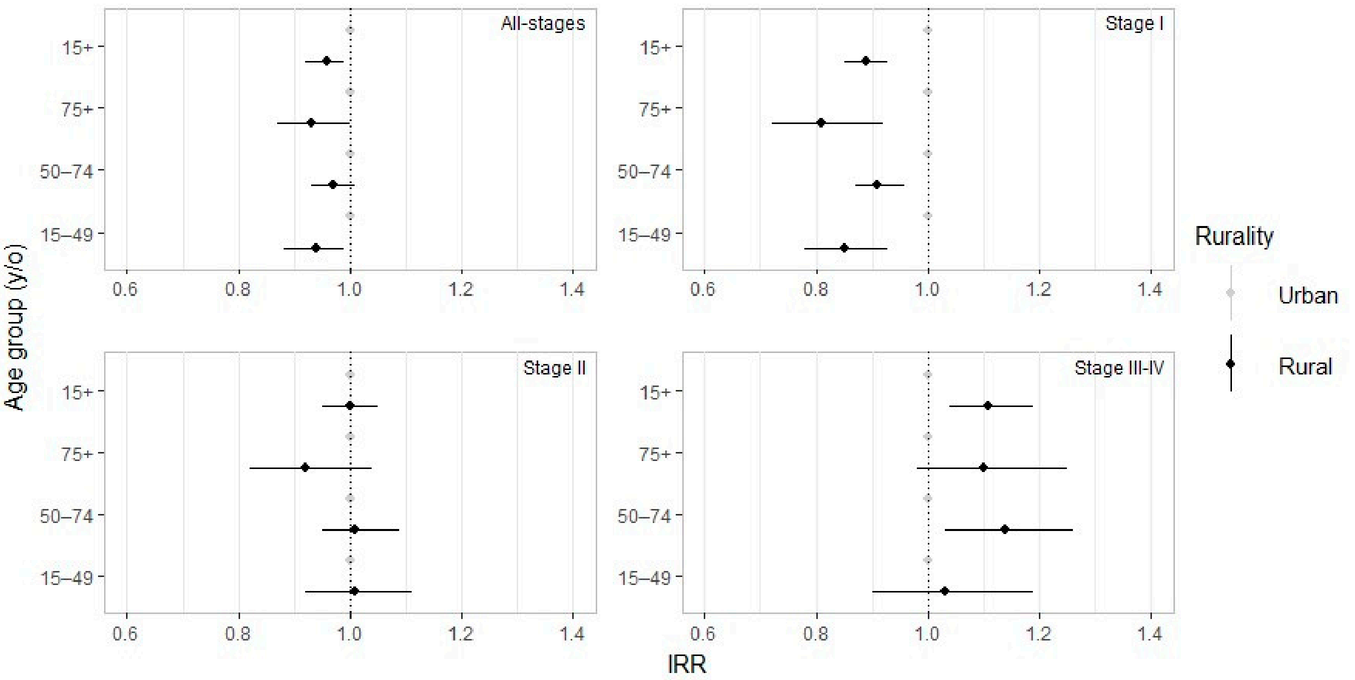

Supplement: Supplementary file 1 [file cancers-16-01701-s001.zip › cancers-2931593-supplementary.pdf]
